# Genetically predicted obesity and risk of hip osteoarthritis

**DOI:** 10.1007/s40519-023-01538-3

**Published:** 2023-02-15

**Authors:** Jiaqin Yuan, Dejie Wang, Yaqiang Zhang, Qiang Dou

**Affiliations:** 1 Department of Orthopedics, Department of Orthopedics, The Second People’s Hospital of Yibin, Sichuan, 644000 China; 2 Health Management Center of Shandong Electric Power Central Hospital, Jinan, 250000 China; 3 Department of Orthopedics, The 940 Hospital of Joint Logistics Support Force of Chinese People’s Liberation Army, Lanzhou, 730050 China

**Keywords:** Mendelian randomization, Obesity, Body mass index, Hip osteoarthritis, Causal effect

## Abstract

**Objectives:**

To determine the causal association between genetically predicted obesity and the risk of hip osteoarthritis.

**Methods:**

We performed two-sample Mendelian randomization (MR) analysis to analyze the association between body mass index (BMI) and hip osteoarthritis using pooled-level genome-wide association study (GWAS) data. The inverse variance weighted (IVW), MR‒Egger, and weighted median methods were used to estimate the causal association. In addition, we applied the MR Steiger filtering method, MR robust adjusted profile score (MR.RAPS) methods, and the MR Pleiotropy RESidual Sum and Outlier (MR-PRESSO) global test to examine and address potential horizontal pleiotropy.

**Results:**

We found a causal relationship between genetically predicted BMI and the risk of hip osteoarthritis by the IVW method [OR = 1.45, 95% confidence interval (CI) = 1.04–2.00, *P* = 0.02]. In the sensitivity analysis, the results of the MR‒Egger and weighted median methods revealed similar estimations but with a wide CI with lower precision. The funnel plot, MR–Egger intercept, and MR-PRESSO all indicated the absence of a directional pleiotropic effect. In addition, no heterogeneity was observed in the present analysis. Therefore, the result of IVW is most suitable and reliable for the present MR analysis.

**Conclusion:**

There is a causal relationship between obesity and a higher risk of hip osteoarthritis, suggesting that weight management may be an intervention for the prevention and management of hip osteoarthritis.

**Level of evidence:**

Bioinformatics, Basic science.

**Supplementary Information:**

The online version contains supplementary material available at 10.1007/s40519-023-01538-3.

## Introduction

Osteoarthritis is a complex joint-related degenerative chronic disease and one of the top ten disabling diseases, with high incidence [1]. It is estimated that approximately 240 million people worldwide suffer from osteoarthritis, with a prevalence of 10% in males and 18% in females over the age of 60 years [2]. With a rapid increase in the older population, the number of osteoarthritis cases may increase by 50% in the next 20 years [3]. As an important structure for weight-bearing and daily activities, the hip joint is prone to lesions leading to osteoarthritis. Radiographic evidence of hip osteoarthritis is found in nearly 5% of people over the age of 65, and the estimated lifetime risk of symptomatic hip osteoarthritis is approximately 25% [4]. As a very large and growing health burden, osteoarthritis has a significant impact on individuals and health care systems, substantial socioeconomic costs, and will result in a worldwide public health crisis [5].

According to the World Health Organization's 2020 research data, the current number of obese people in the world is three times that in 1975, and approximately 39% of adults are overweight or obese. For adults over the age of 18 years, more than 1 in 3 are overweight, and 1 in 10 are obese. By 2050, 60% of adult men, 50% of adult women, and 25% of children (under 16 years) are predicted to be obese [6]. Epidemiological studies show that there are approximately 2.1 billion obese people in the world. This number continues to rise [7].

Research results [8, 9] show that overweight and obesity are important risk factors for osteoarthritis. From a weight-bearing perspective, the weight load caused by overweight and obesity may put an excess load on the hip joint and lead to hip osteoarthritis [10]. From a nonweight-bearing point of view, being overweight and obese can lead to metabolic changes in the body, and these metabolic abnormalities may lead to abnormal metabolism of articular cartilage and eventually lead to hip osteoarthritis [10]. An epidemiological study has shown that a 5-unit increase in BMI is associated with an increased risk of hip osteoarthritis (RR = 1.11; 95% CI: 1.07, 1.16) [11]. However, epidemiological studies may suffer from measurement error, uncontrolled confounding factors, and reverse causality. Ultimately, the results may be subject to various biases. Therefore, a study is needed with a design that can avoid or reduce some of the biases to further demonstrate the relationship between obesity and hip osteoarthritis.

Mendelian randomization (MR) is a potential causal inference method that uses genetic variation as an instrumental variable to obtain the effect of exposure factors on outcomes from observational data [12]. MR can reduce the effects of non-measurement errors or confounding factors while avoiding reverse causality through Mendelian inheritance laws [12]. Therefore, this study aimed to use MR to demonstrate whether obesity and hip osteoarthritis are causally related.

## Methods

### MR design

**Fig. 1:**
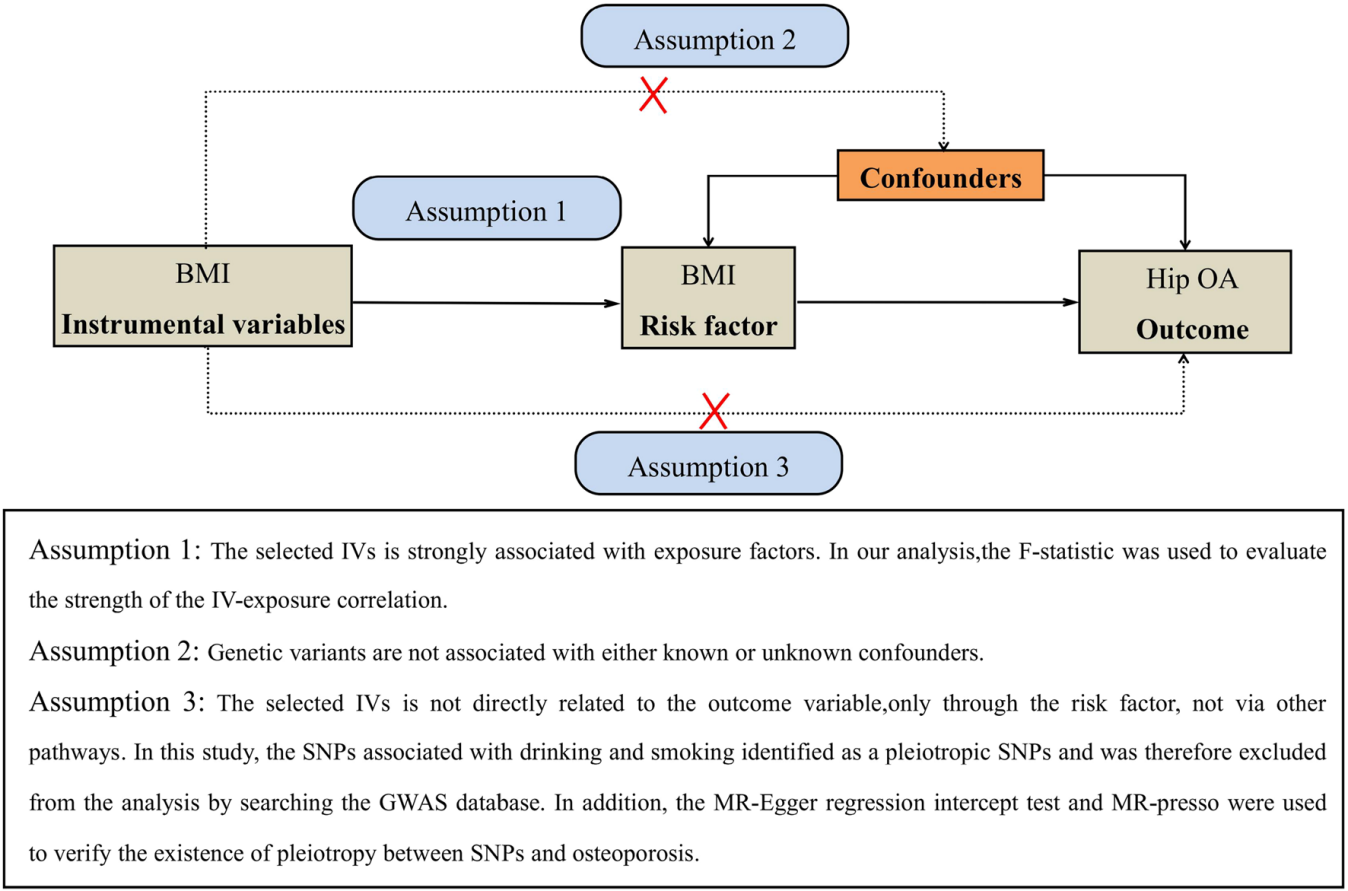
Design flow chart for the MR study

For this study, two-sample MR was used to explore the causal relationships between genetic risk factors (e.g., body mass index) and genetic outcomes (e.g., hip osteoarthritis). This strategy uses publicly available aggregated data on SNP exposure and SNP outcome associations from different data sources, which has been shown to yield accurate estimates of the causal effect of exposure on outcomes [13]. In our study, single nucleotide polymorphisms (SNPs) were defined as instrumental variables (IVs). The MR method needs to satisfy three basic IVs assumptions: (1) genetic variation is strongly associated with exposure; (2) genetic variation should not be associated with any potential confounders; and (3) IVs must not affect outcomes independently of their effect on exposure (Fig. 1). In addition, other assumptions, such as linearity and the absence of statistical interaction, should also be satisfied [14]. We included SNPs that were significant at the genome-wide level (*P* < 5 × 10^−8^) and had a minor allele frequency > 0.01. Then these SNPs were clumped based on linkage disequilibrium (window size = 10,000 kb and *r*^2^ < 0.001). Linkage disequilibrium levels were estimated from the 1000 Genomes Project based on European samples [15]. We then evaluated the power of the remaining SNPs using the F statistics (*F* = (β/SE)^2^) and removed weak instrumental variables (F statistics < 10) [16].

### Data sources

We extracted IVs for BMI from the Genetic Investigation of Anthropometric Traits (GIANT) consortium. We identified 78 SNPs associated with BMI through a meta-analysis of 339,224 individuals of mixed ancestry. Although the GIANT consortium was performed in individuals of mixed ancestry, only SNPs in populations of European ancestry that achieved genome-wide significance (*P* < 5 × 10^−8^) were used in our study [17]. We used the publicly available summary statistic datasets of a GWAS for hip OA from the individuals included in the NHGRI-EBI (total *n* = 11,989, hip OA: 2396, control: 9593) (https://www.ebi.ac.uk/gwas/) as the outcome. More detailed information on the UK Biobank has been described elsewhere [18].

## Statistical analysis

MR analysis was performed by R software (version 4.2.0, http://www.r-project.org) and the “TwoSampleMR” package (version 0.5.6) [19]. MR-PRESSO and RAPS were performed using the R packages "MRPRESSO" and "MR.raps", respectively. Calculation of statistical power for Mendelian randomization was conducted using mRnd (https://cnsgenomics.shinyapps.io/mRnd/).

Inverse variance weighting (IVW) used meta-analysis to combine the Wald ratios of causal effects for each SNP [20, 21]. Then the MR‒Egger [22]and weighted median [23] methods were used as a complement to IVW. Different methods adapted to different validity assumptions were applied to obtain MR estimates. The application of IVW is based on the premise that all SNPs are valid instrumental variables. Therefore, this method can obtain accurate estimation results. MR‒Egger assesses directional pleiotropy for instrumental variables, where the intercept can be interpreted as an estimate of the average pleiotropy of genetic variation. The weighted median has the advantage of maintaining higher precision (smaller standard deviation) than MR‒Egger analysis. In the presence of horizontal pleiotropy, the weighted median provides a consistent estimate even if 50% of the genetic variants are invalid IVs [24]. If the following three conditions were met, we believed that there was a significant causal association between cystatin C levels and osteoporosis: 1) there was a significant difference in the IVW method (*P* < 0.05), 2) the estimation directions of the IVW, weighted median, and MR–Egger methods were consistent, and 3) neither the MR–Egger intercept test nor the MR-PRESSO global test was significant (*P* > 0.05).

The causal direction of each extracted SNP to the exposure and outcome was tested using MR Steiger filtering [25]. This method calculates the variance explained in exposure and results from the instrumental SNP and tests whether the variance in the results is less than that in the exposure. "TRUE" MR Steiger results indicate causality in the expected direction, while "FALSE" results indicate causality in the opposite direction. We excluded SNPs with 'FALSE' results, meaning they showed evidence of a major effect on the outcome rather than exposure. In addition, a recently proposed method called MR Robust Adjusted Profile Score (MR.RAPS) was performed to make the results more robust [25]. MR.RAPS takes into account measurement errors in SNP exposure effects, is impartial when using weak instruments, can correct for pleiotropy with robust adjusted contour scores, and is robust to both systemic and heterogeneous pleiotropy [25].

Heterogeneity between SNPs was assessed using Cochran's Q statistic and funnel plots. Horizontal pleiotropy was detected using the MR‒Egger intercept [22] method and the MR pleiotropy residual sum and outlier test (MR-PRESSO) [26] method. If outliers were detected, they were removed, and we re-evaluated the MR causal estimates. If heterogeneity persisted after removal, a random-effects IVW model was implemented, which was less prone to bias toward weaker SNP exposure associations. Finally, leave-one-out analysis was used to assess the stability of effect sizes and to determine whether the results were driven by any single SNP.

## Data availability and ethical approval

All of our data were obtained from published studies, which were supported by the institutional review committee, and informed consent was obtained from the participants in their original research [27, 28]. Thus, further sanctions were not needed.

## Results

### Causal associations between BMI and hip OA

We first extracted 78 significant genome-wide (*P* < 5 × 10^−8^) and independently inherited SNPs associated with BMI. We also removed palindromic SNPs when harmonizing the effect of IVs, excluding 17 SNPs with false causal directions identified by MR Steiger filtering. Finally, we identified 61 SNPs (Table S1) as instrumental variables for BMI. The collectively explained variance in BMI was 2.0%. The strength of the selected single IVs had an F-statistic value between 30 and 716. Therefore, no SNPs were weak instrumental variables. In this study, because of the relatively large sample size of the outcome databases, a type I error of 1% and statistical power of 0.95 were considered. The causal effects of each SNP on hip osteoarthritis are shown in Figures S1 and S2.

### Mendelian randomization results

**Fig. 2:**
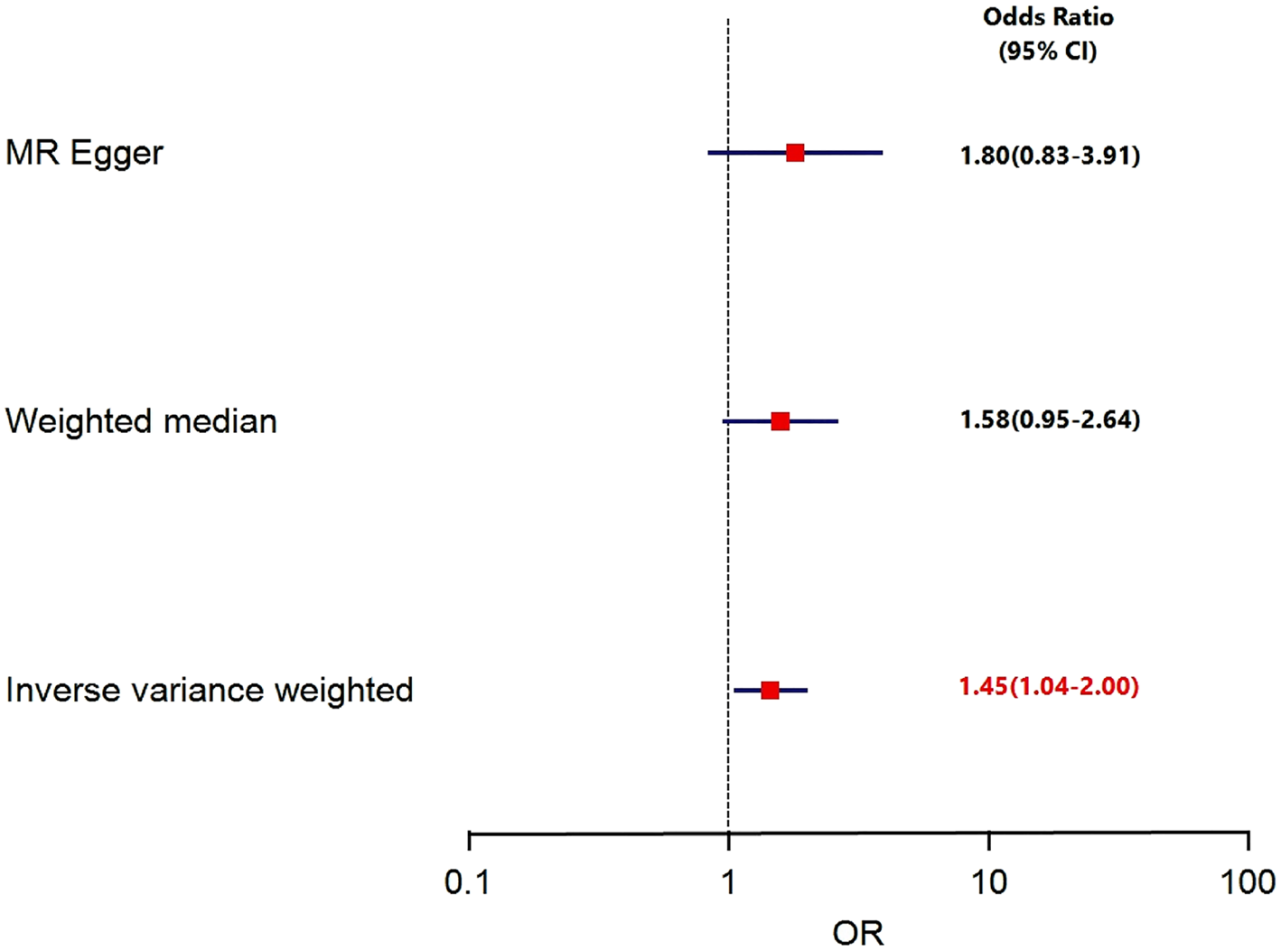
Forest plot to visualize causal effect of BMI on hip osteoarthritis risk by three methods

IVW, MR‒Egger, and weighted median regression were used to estimate the causal relationship between genetically predicted BMI and hip osteoarthritis (Fig. 2; Table S2). Using the main IVW approach, genetically predicted BMI was found to be positively associated with the risk of hip osteoarthritis [OR = 1.45, 95% CI = 1.04–2.00, *P* = 0.02]. Weighted median regression also showed directionally similar estimates [OR = 1.58, 95% CI = 0.95–2.64, *P* = 0.08]. The MR–Egger method showed no statistical significance [OR = 1.80, 95% CI = 0.83–3.91, *P* = 0.14]. Fortunately, the estimation directions of the three methods were consistent. Finally, we used MR to reduce the effect of potentially weak instrumental variables on the robustness of the results. The RAPS test showed that genetically predicted BMI was still positively associated with the risk of hip osteoarthritis [OR = 1.45, 95% CI = 1.03–2.04, *P* = 0.03].

## Sensitivity analysis

**Fig. 3:**
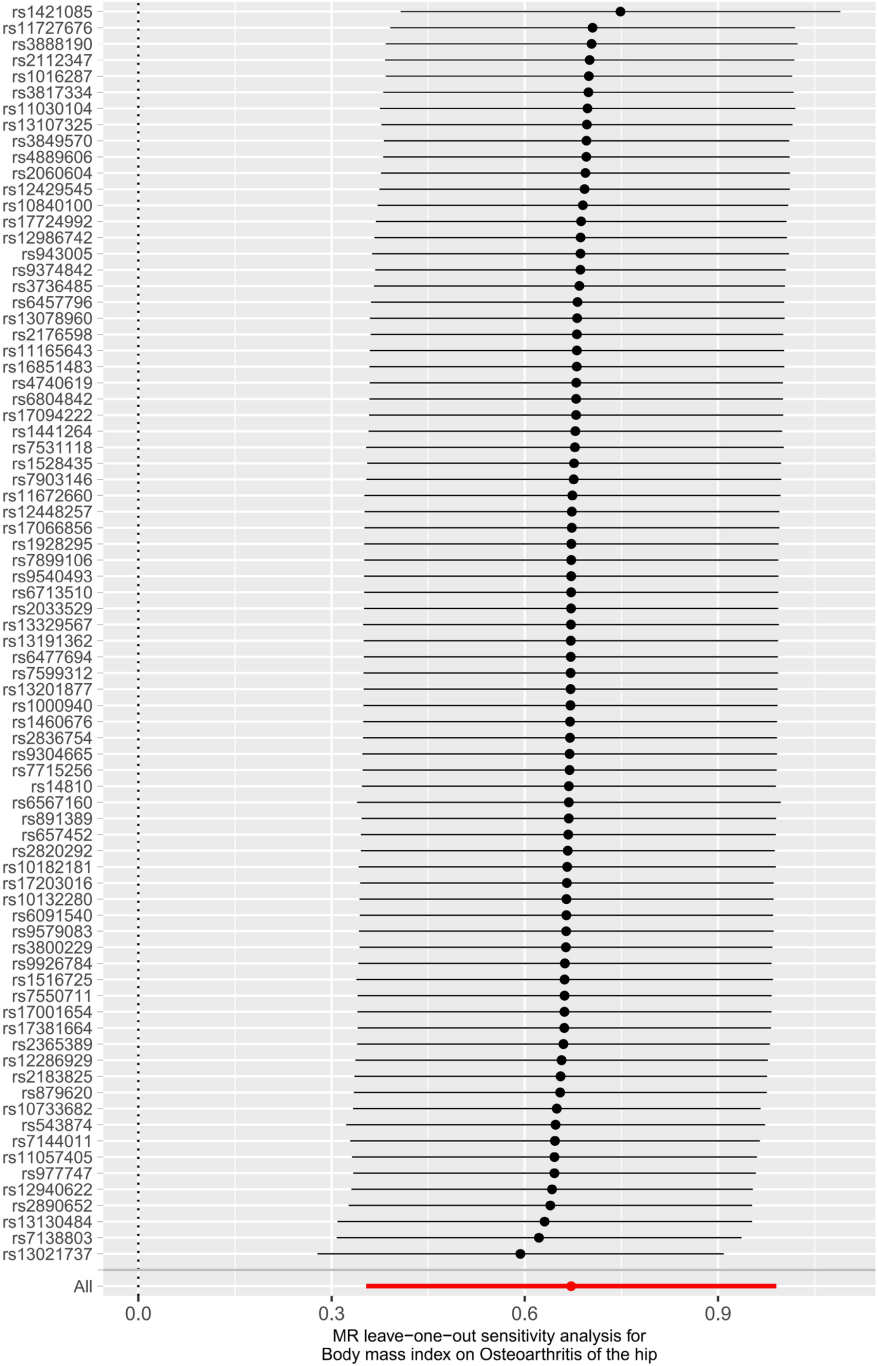
Sensitivity analysis of the effect of BMI on hip osteoarthritis

Several sensitivity analyses were used to examine and correct for the presence of pleiotropy in causal estimates. Cochran's Q test (Table S2) and funnel plot (Figure S3) showed no evidence of heterogeneity or asymmetry between these SNPs in the causal effect between BMI and hip osteoarthritis. In our study, there was no evidence that the results were affected by genetic pleiotropy according to the MR‒Egger intercept test (Table S2). For each SNP, no potential pleiotropy was found using the MR-PRESSO global test (Table S2). We verified the impact of each SNP on the overall causal estimate by conducting leave-one-out analysis. As shown in Fig. 3, we systematically performed the MR analysis again on the remaining SNPs after removing each SNP. The results remained consistent, suggesting that the results of all SNPs made the causal inference significant. This also indicates that there was no dominant SNP in BMI and hip osteoarthritis, and the MR results were valid.

## Discussion

Recent studies [29–32] have extensively examined the relationship between obesity and osteoarthritis. However, the evidence in the available studies is limited by showing observational correlations, and the results may be influenced by confounding factors. This study aimed to elucidate the causal effects of obesity on hip osteoarthritis. In this study, we used MR to investigate the association between obesity and the risk of hip osteoarthritis based on existing GWAS. Our results suggest that genetically predicted obesity is significantly associated with an increased risk of hip osteoarthritis. The risk of hip osteoarthritis increased by 96% for each one standard deviation increase in BMI.

In our study, statistical significance was assessed using the two-sample MR method, with summary statistics from the largest study on BMI (*n* = 339,224) and hip osteoarthritis (up to *n* = 2396 hip osteoarthritis cases and 9,593 controls). Our results show a causal relationship between obesity and hip osteoarthritis. To check the reliability of our findings and minimize potential pleiotropic effects, we performed a series of sensitivity analyses. The results were robust when sensitivity analyses were performed using MR-PRESSO, MR‒Egger regression intercept, and leave-one-out analysis. We searched in Phenoscanner and the GWAS catalog and confirmed no evidence of pleiotropy, which suggests that our results are reasonable. Although we require the F value of the instrumental variable to be greater than 10 to eliminate weak instrumental variables, to avoid potential weak instrumental variables from affecting the results, we conducted MR.RAPS analysis, and the results were not affected. Our findings are also similar to previous results [33]. The MR Steiger test further showed that there was no evidence of reverse causality in our study. Our findings are also similar to previous findings. However, in the previous MR study [33], they used GWAS from mixed populations, which is a very large limitation, as they describe in the discussion. Therefore, it is necessary for us to perform MR analysis in a pure European population. Another limitation is the high heterogeneity of their studies and the substantial horizontal pleiotropy (*P* < 0.001) using the MR-PRESSO test. In addition, they did not remove IVs that exhibited reverse causality. Of course, our study is also different from the previous study. Our findings were specific to osteoarthritis of the hip, not the knee. We consider specific joint sites to be more specific than general osteoarthritis. In summary, we believe that our study provides a valuable contribution.

Osteoarthritis is an irreversible chronic disease characterized by two main pathological features: articular cartilage damage and subchondral sclerosis, which involve the entire joint, including cartilage degeneration, bone remodeling, osteophyte formation, and synovial inflammation, resulting in joint pain, stiffness, and loss of normal function. Inflammation of the synovium results in excessive production of synovial fluid, swelling of the joints, and inhibition and chronic disuse of the muscles that connect the joints, eventually leading to muscle wasting and disability. Pain is the main symptom and the main driver of clinical decision-making and health service use [3]. Risk factors for osteoarthritis include fixed (e.g., age, sex) and modifiable (e.g., overweight or obesity, physical activity) factors, at least in principle. To date, the link between being overweight or obese and osteoarthritis has been consistently demonstrated in osteoarthritis of the knee [34]. However, data on hip joints, obesity, and osteoarthritis have been inconsistent. In a population survey from the United States [35], obesity was not associated with unilateral hip osteoarthritis. Similarly, in a prospective study of the Finnish population [36], BMI was not a predictor of risk factors for hip osteoarthritis. Similarly, in a prospective study of the Finnish population, BMI was not a predictor of risk factors for hip osteoarthritis. In contrast, the study by Holliday et al. [37] showed that each SD increase in BMI was associated with a 65% increased risk of hip osteoarthritis (*P* < 0.001). A prospective cohort study of more than 120,000 people found that only higher BMI and older age were associated with an increased risk of osteoarthritis [38]. In particular, women with the highest BMI had twice the risk of hip replacement surgery than women with the lowest BMI. A meta-analysis based on 14 studies [11] showed that BMI was significantly positively associated with hip osteoarthritis risk. Thus, there is increasing evidence of deleterious effects of obesity on hip osteoarthritis.

The mechanism of action of obesity on hip osteoarthritis remains unclear. At present, it is believed that obesity mainly causes hip osteoarthritis as a result of weight-bearing and nonweight-bearing aspects. The cause of the onset and progression of hip osteoarthritis may be abnormal mechanical loading due to weight gain in weight-bearing joints. The main function of articular cartilage is to provide the joint with a smooth surface with a low coefficient of friction and to facilitate the transmission of loads during joint motion [39]. When the cartilage load exceeds the critical value, articular cartilage will be irreversibly damaged. At first, articular cartilage swelling and proteoglycan loss are significantly increased, hypertrophic chondrocytes are activated, and then chondrocyte necrosis and cartilage thinning occur gradually [40]. In addition, supraphysiological loading can lead to increased proinflammatory cytokines in articular chondrocytes and synovial cells, thereby promoting a vicious cycle of synovial joint pathological changes. Several studies [41–43] have shown no increase in subchondral sclerosis with increasing BMI, confirming that the increase in symptoms in obese patients cannot be explained by more structural joint damage. Therefore, obesity may be involved in the pathogenesis of hip osteoarthritis not only by increasing mechanical load but also via local inflammation. Schelbergen et al. [44] showed that the activated macrophage-associated alarmin S100A/S100A9 induces overexpression and activation of matrix metalloproteinases, leading to cartilage matrix remodeling and osteophyte formation. Then abnormal production and secretion of adipokines (including leptin, adiponectin, resistin, etc.) are also thought to play an important role in the association between obesity and systemic inflammation. For example, the consumption of a high-fat diet led to a significant increase in leptin, which was positively associated with OA-related cartilage damage, osteophytes, and increased infrapatellar fat pad size [45]. To date, the specific mechanism of action of obesity-induced hip osteoarthritis remains unclear.

### Strength and limits

Our study has several major strengths. The main advantage is the MR design, which estimates the causal effect of obesity on hip osteoarthritis without interference from residual confounding or reverse causality. Regarding causal inference from observational studies, no matter how well designed the epidemiological study is and how accurate the measurements are, potential, unmeasurable confounders cannot be eliminated. We performed MR Steiger filtering to exclude all SNPs that primarily affected hip osteoarthritis but not BMI. We also performed the MR.RAPS method, which can provide robust inferences for our MR analysis with many weak IVs. Finally, we also used various sensitivity analysis methods to ensure the robustness of the results.

Of course, our MR study had several limitations. First, the available data we use are summary-level statistics, not individual-level statistics. Second, our MR analysis is based on individuals of European ancestry; therefore, validation for individuals of other ancestries is needed. Finally, exposure and outcome studies used in two-sample MR analyses should not involve overlapping participants. We were unable to estimate the degree of overlap in the studies. To avoid this, we used GWAS data from different Consortiums while using powerful tools to minimize overlap (e.g., F statistics much greater than 10).

### What is already known on this subject?

Obesity has been reported to be one of the major risk factors for osteoarthritis. However, the relationship between obesity and hip OA remains controversial in many observational studies.

### What does this study add?

This MR study shows a positive association between obesity and hip osteoarthritis. Obesity may also be a major risk factor for hip osteoarthritis.

## Conclusion

In conclusion, there is a causal relationship between increased obesity and hip osteoarthritis. Our research supports weight management as an intervention for the prevention and management of hip osteoarthritis.

## Supplementary Information

Below is the link to the electronic supplementary material.

Supplementary file1 (DOC 1649 KB)
