# Supplementary material for: Genetically predicted obesity and risk of hip osteoarthritis
Source: Eat Weight Disord. 2023 Feb 15;28(1):11. doi: 10.1007/s40519-023-01538-3 (PMC9931853; doi:10.1007/s40519-023-01538-3)
Supplement: Supplementary file 1 — Supplementary file1 (DOC 1649 KB) [file 40519_2023_1538_MOESM1_ESM.doc]

**Table S1** | List of genetic instruments for BMI and log odds ratios of hip osteoarthritis risk by each instrumental SNPs (GWAS significance with p < 5 × 10^−8^ and linkage disequilibrium. threshold with R^2^ < 0.001)

| No. | SNPs | Chr. | EA/NEA | EAF | Association with BMI | | | | Association with hip OA | | |
| --- | --- | --- | --- | --- | --- | --- | --- | --- | --- | --- | --- |
|  |  |  |  |  | β | SE | P-value | F | β | SE | P-value |
| 1 | rs1000940 | 17 | G/A | 0.23 | 0.018 | 0.0033 | 1.81E-08 | 31 | 0.016 | 0.04 | 0.66 |
| 2 | rs10132280 | 14 | A/C | 0.33 | -0.022 | 0.0033 | 1.40E-11 | 45 | -0.033 | 0.04 | 0.35 |
| 3 | rs10182181 | 2 | G/A | 0.50 | 0.031 | 0.0029 | 8.07E-26 | 114 | 0.030 | 0.03 | 0.36 |
| 4 | rs10840100 | 11 | G/A | 0.73 | 0.021 | 0.0030 | 6.67E-12 | 47 | -0.030 | 0.03 | 0.37 |
| 5 | rs11030104 | 11 | G/A | 0.20 | -0.042 | 0.0037 | 6.66E-30 | 126 | 0.014 | 0.04 | 0.72 |
| 6 | rs11165643 | 1 | T/C | 0.58 | 0.022 | 0.0030 | 1.43E-13 | 54 | -0.004 | 0.03 | 0.90 |
| 7 | rs11672660 | 19 | T/C | 0.18 | -0.034 | 0.0038 | 7.91E-19 | 80 | -0.019 | 0.04 | 0.65 |
| 8 | rs12429545 | 13 | A/G | 0.10 | 0.032 | 0.0044 | 3.15E-13 | 54 | -0.047 | 0.05 | 0.34 |
| 9 | rs12448257 | 16 | A/G | 0.23 | 0.025 | 0.0037 | 3.90E-11 | 44 | 0.014 | 0.04 | 0.73 |
| 10 | rs12986742 | 2 | C/T | 0.50 | 0.021 | 0.0036 | 8.92E-09 | 33 | -0.019 | 0.03 | 0.56 |
| 11 | rs13078960 | 3 | G/T | 0.18 | 0.029 | 0.0038 | 1.42E-14 | 58 | -0.003 | 0.04 | 0.94 |
| 12 | rs13107325 | 4 | T/C | 0.12 | 0.047 | 0.0066 | 1.06E-12 | 51 | -0.053 | 0.06 | 0.39 |
| 13 | rs13130484 | 4 | T/C | 0.43 | 0.040 | 0.0030 | 8.01E-41 | 176 | 0.074 | 0.03 | 0.02 |
| 14 | rs13191362 | 6 | G/A | 0.20 | -0.029 | 0.0047 | 1.09E-09 | 37 | -0.021 | 0.05 | 0.67 |
| 15 | rs13201877 | 6 | G/A | 0.08 | 0.024 | 0.0043 | 4.28E-08 | 30 | 0.019 | 0.05 | 0.68 |
| 16 | rs13329567 | 15 | T/C | 0.22 | -0.031 | 0.0035 | 1.53E-18 | 77 | -0.021 | 0.04 | 0.59 |
| 17 | rs1421085 | 16 | C/T | 0.45 | 0.080 | 0.0030 | 2.17E-158 | 716 | 0.014 | 0.03 | 0.66 |
| 18 | rs1441264 | 13 | A/G | 0.55 | 0.017 | 0.0031 | 2.96E-08 | 31 | -0.006 | 0.03 | 0.85 |
| 19 | rs1460676 | 2 | C/T | 0.22 | 0.021 | 0.0038 | 4.98E-08 | 30 | 0.021 | 0.04 | 0.64 |
| 20 | rs14810 | 19 | G/C | 0.68 | 0.018 | 0.0033 | 1.92E-08 | 31 | 0.023 | 0.03 | 0.51 |
| 21 | rs1516725 | 3 | C/T | 0.91 | 0.045 | 0.0044 | 1.39E-24 | 104 | 0.053 | 0.05 | 0.27 |
| 22 | rs1528435 | 2 | T/C | 0.58 | 0.018 | 0.0030 | 4.77E-09 | 34 | -0.001 | 0.03 | 0.98 |
| 23 | rs16851483 | 3 | T/G | 0.09 | 0.048 | 0.0075 | 1.85E-10 | 41 | -0.001 | 0.06 | 0.99 |
| 24 | rs17001654 | 4 | G/C | 0.16 | 0.030 | 0.0052 | 5.03E-09 | 34 | 0.049 | 0.04 | 0.26 |
| 25 | rs17066856 | 18 | C/T | 0.13 | -0.037 | 0.0050 | 2.00E-13 | 55 | -0.021 | 0.06 | 0.71 |
| 26 | rs17094222 | 10 | C/T | 0.21 | 0.025 | 0.0037 | 2.19E-11 | 45 | -0.004 | 0.04 | 0.91 |
| 27 | rs17203016 | 2 | G/A | 0.20 | 0.021 | 0.0038 | 3.41E-08 | 31 | 0.037 | 0.04 | 0.36 |
| 28 | rs17381664 | 1 | C/T | 0.43 | 0.020 | 0.0031 | 4.57E-11 | 42 | 0.039 | 0.03 | 0.24 |
| 29 | rs17724992 | 19 | G/A | 0.31 | -0.020 | 0.0034 | 7.79E-09 | 33 | 0.033 | 0.04 | 0.36 |
| 30 | rs1928295 | 9 | C/T | 0.43 | -0.018 | 0.0029 | 4.32E-10 | 39 | -0.011 | 0.03 | 0.73 |
| 31 | rs2033529 | 6 | G/A | 0.26 | 0.018 | 0.0032 | 1.45E-08 | 33 | 0.013 | 0.04 | 0.72 |
| 32 | rs2060604 | 8 | C/T | 0.44 | -0.020 | 0.0030 | 9.46E-12 | 46 | 0.038 | 0.03 | 0.25 |
| 33 | rs2112347 | 5 | G/T | 0.38 | -0.025 | 0.0030 | 1.96E-17 | 72 | 0.038 | 0.03 | 0.25 |
| 34 | rs2176598 | 11 | C/T | 0.80 | -0.019 | 0.0033 | 3.47E-08 | 31 | 0.018 | 0.04 | 0.64 |
| 35 | rs2820292 | 1 | C/A | 0.51 | 0.018 | 0.0029 | 5.45E-10 | 39 | 0.026 | 0.03 | 0.43 |
| 36 | rs2836754 | 21 | C/T | 0.65 | 0.017 | 0.0030 | 1.60E-08 | 32 | 0.017 | 0.03 | 0.62 |
| 37 | rs3736485 | 15 | G/A | 0.58 | -0.016 | 0.0029 | 4.52E-08 | 30 | 0.027 | 0.03 | 0.41 |
| 38 | rs3817334 | 11 | T/C | 0.45 | 0.026 | 0.0030 | 1.17E-17 | 73 | -0.033 | 0.03 | 0.32 |
| 39 | rs3888190 | 16 | A/C | 0.36 | 0.031 | 0.0030 | 3.45E-25 | 107 | -0.028 | 0.03 | 0.40 |
| 40 | rs4740619 | 9 | C/T | 0.47 | -0.017 | 0.0029 | 6.36E-09 | 34 | 0.010 | 0.03 | 0.75 |
| 41 | rs543874 | 1 | G/A | 0.27 | 0.050 | 0.0037 | 2.29E-40 | 180 | 0.066 | 0.04 | 0.10 |
| 42 | rs6091540 | 20 | T/C | 0.28 | -0.019 | 0.0033 | 2.14E-08 | 31 | -0.035 | 0.04 | 0.33 |
| 43 | rs6457796 | 6 | C/T | 0.26 | 0.021 | 0.0033 | 2.54E-10 | 40 | -0.014 | 0.04 | 0.71 |
| 44 | rs6477694 | 9 | T/C | 0.64 | -0.017 | 0.0030 | 1.70E-08 | 32 | -0.013 | 0.03 | 0.70 |
| 45 | rs6567160 | 18 | C/T | 0.28 | 0.056 | 0.0035 | 6.68E-59 | 258 | 0.042 | 0.04 | 0.27 |
| 46 | rs657452 | 1 | G/A | 0.58 | -0.023 | 0.0031 | 2.12E-13 | 54 | -0.025 | 0.03 | 0.46 |
| 47 | rs6713510 | 2 | A/G | 0.48 | 0.016 | 0.0029 | 1.97E-08 | 32 | 0.011 | 0.03 | 0.74 |
| 48 | rs6804842 | 3 | G/A | 0.58 | 0.018 | 0.0030 | 8.02E-10 | 37 | -0.008 | 0.03 | 0.81 |
| 49 | rs7531118 | 1 | C/T | 0.61 | 0.033 | 0.0030 | 1.88E-28 | 122 | 0.014 | 0.03 | 0.67 |
| 50 | rs7550711 | 1 | T/C | 0.03 | 0.066 | 0.0087 | 5.06E-14 | 57 | 0.108 | 0.10 | 0.27 |
| 51 | rs7599312 | 2 | A/G | 0.29 | -0.021 | 0.0033 | 4.73E-11 | 42 | -0.017 | 0.04 | 0.65 |
| 52 | rs7715256 | 5 | T/G | 0.55 | -0.017 | 0.0029 | 8.85E-09 | 34 | -0.018 | 0.03 | 0.58 |
| 53 | rs7899106 | 10 | G/A | 0.05 | 0.038 | 0.0067 | 1.27E-08 | 32 | 0.025 | 0.07 | 0.74 |
| 54 | rs7903146 | 10 | T/C | 0.25 | -0.024 | 0.0033 | 1.10E-12 | 51 | -0.006 | 0.04 | 0.86 |
| 55 | rs891389 | 18 | T/C | 0.33 | 0.021 | 0.0037 | 1.62E-08 | 32 | 0.023 | 0.03 | 0.50 |
| 56 | rs9304665 | 19 | A/T | 0.70 | 0.024 | 0.0043 | 1.59E-08 | 32 | 0.023 | 0.04 | 0.56 |
| 57 | rs9374842 | 6 | T/C | 0.74 | 0.020 | 0.0034 | 7.20E-09 | 33 | -0.034 | 0.04 | 0.37 |
| 58 | rs943005 | 6 | T/C | 0.10 | 0.044 | 0.0038 | 4.52E-31 | 137 | 0.003 | 0.04 | 0.95 |
| 59 | rs9540493 | 13 | G/A | 0.55 | -0.018 | 0.0031 | 3.95E-09 | 34 | -0.012 | 0.03 | 0.71 |
| 60 | rs9579083 | 13 | C/G | 0.23 | 0.030 | 0.0046 | 1.43E-10 | 41 | 0.040 | 0.04 | 0.34 |
| 61 | rs9926784 | 16 | C/T | 0.21 | -0.025 | 0.0038 | 8.55E-11 | 43 | -0.048 | 0.04 | 0.26 |

Chr. indicates chromosome; EA, effect allele; NEA, non-effect allele; EAF, effect allele frequency; F, F-statistics; OA, hip Osteoarthritis; SE, standard error.

**Table S2** | Two-sample Mendelian randomization estimations showing the effect of BMI on hip osteoarthritis.

| Exposure/outcome | Methods | Odds ratio | 95%CI | P-value | Q-statistics | P_h_ |
| --- | --- | --- | --- | --- | --- | --- |
| BMI/hip OA | Inverse-variance weighted | 1.45 | 1.04–2.00 | 0.02 | 60 | 0.999 |
|  | Weighted median | 1.58 | 0.95–2.64 | 0.08 | - | - |
|  | MR-Egger | 1.80 | 0.83–3.91 | 0.14 | 59 | 0.999 |
|  | MR.RAPS | 1.45 | 1.03–2.04 | 0.03 | - | - |
|  | MR-Egger intercept | 0.993 | 0.97-1.02 | 0.54 | - | - |
|  | MR-presso | 1.45 | 1.14-1.83 | 0.003 | - | - |

P_h_, P-value for heterogeneity.


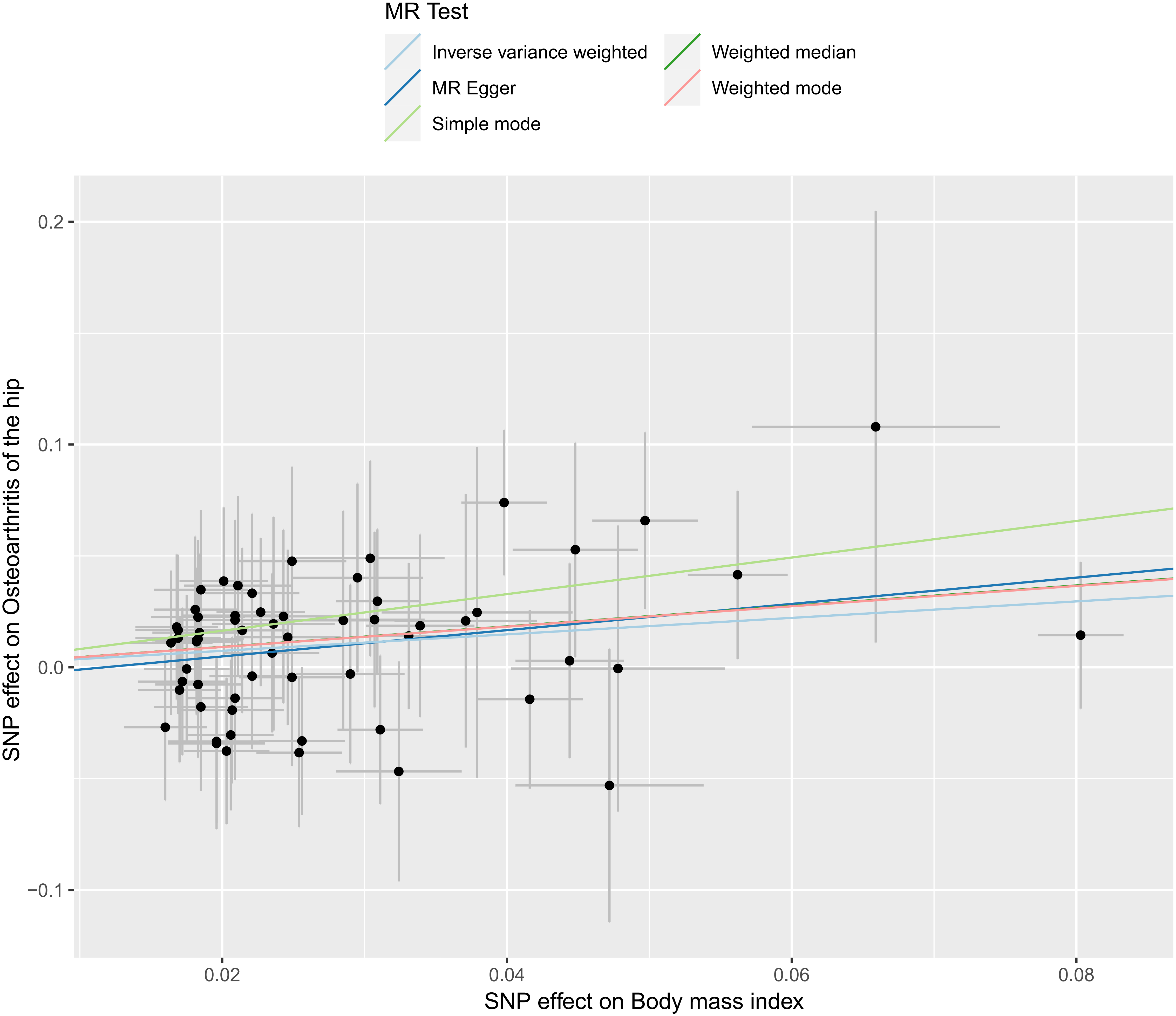


**Figure S1** | Scatter plot to visualize causal effect of BMI on hip osteoarthritis risk.


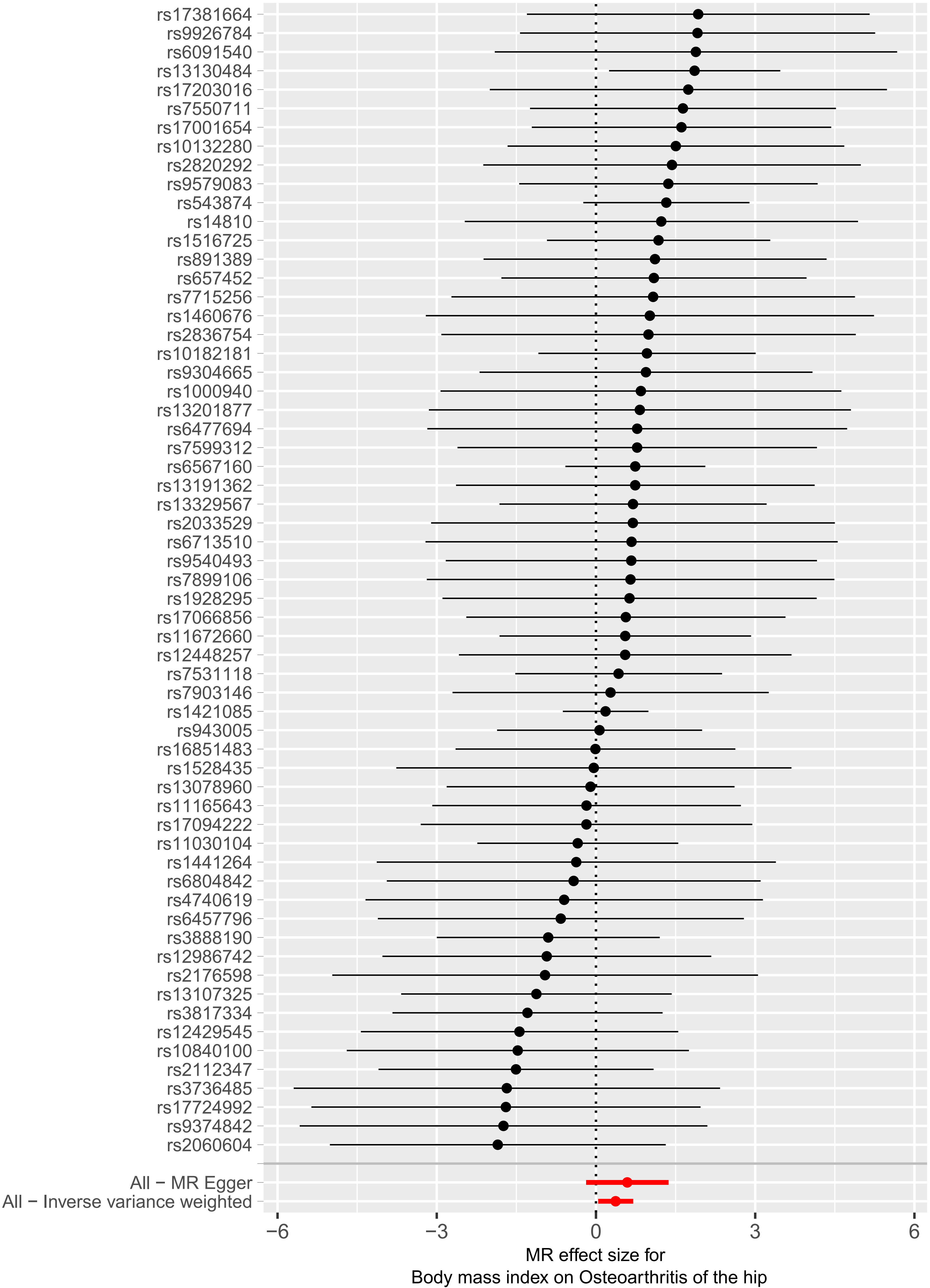


**Figure S2** | Forest plot to visualize causal effect of each single SNP on hip osteoarthritis risk.


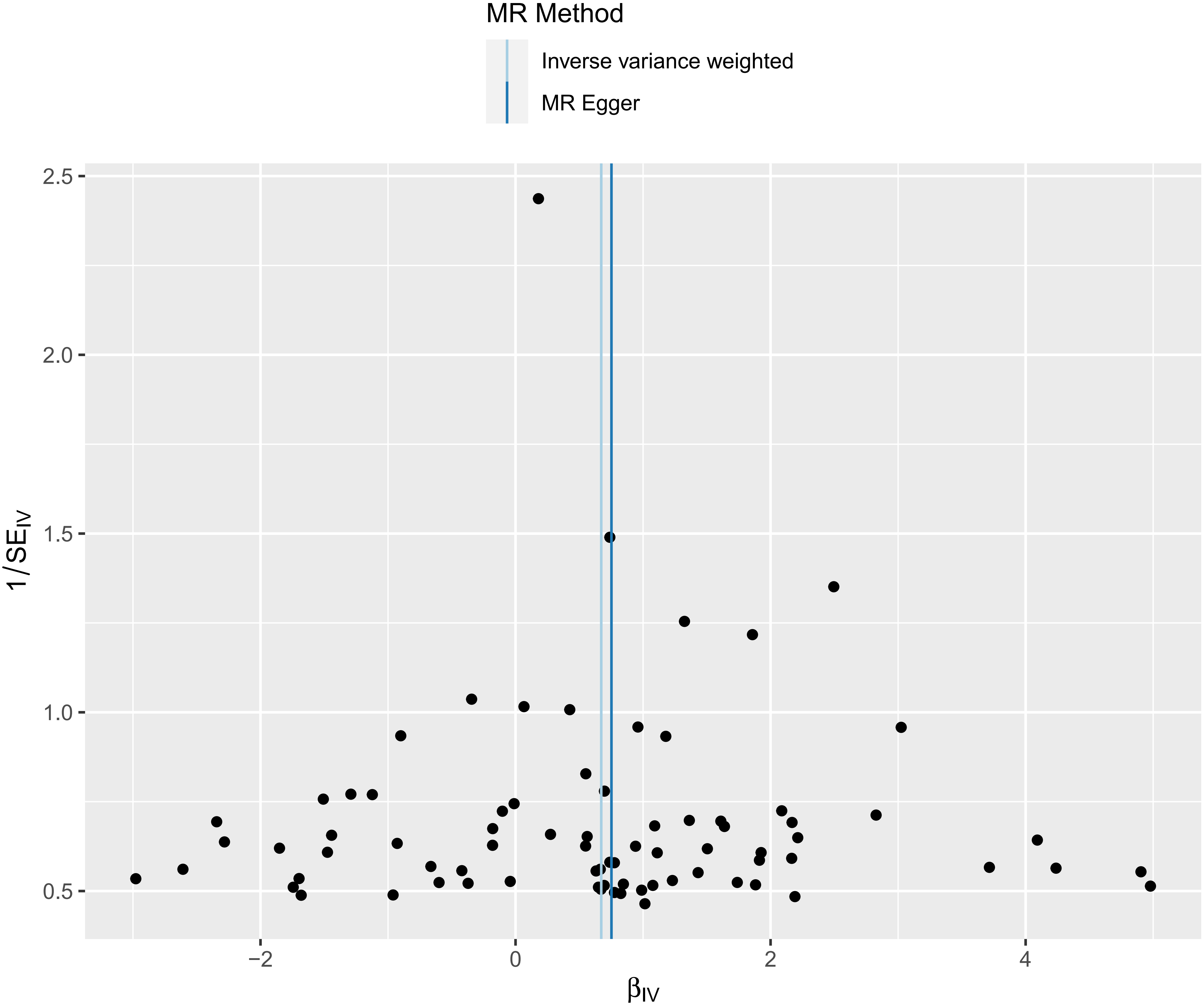


**Figure S3** | Funnel plots to visualize overall heterogeneity of MR estimates for the effect of BMI on hip osteoarthritis.
